# Genome Analysis of Coxsackievirus A4 Isolates From Hand, Foot, and Mouth Disease Cases in Shandong, China

**DOI:** 10.3389/fmicb.2019.01001

**Published:** 2019-05-07

**Authors:** Min Wang, Juan Li, Ming-Xiao Yao, Ya-Wei Zhang, Tao Hu, Michael J. Carr, Sebastián Duchêne, Xing-Cheng Zhang, Zhen-Jie Zhang, Hong Zhou, Yi-Gang Tong, Shu-Jun Ding, Xian-Jun Wang, Wei-Feng Shi

**Affiliations:** ^1^Key Laboratory of Etiology and Epidemiology of Emerging Infectious Diseases in Universities of Shandong, Taishan Medical College, Tai’an, China; ^2^Shandong Provincial Key Laboratory of Communicable Disease Control and Prevention, Institute for Viral Disease Control and Prevention, Shandong Center for Disease Control and Prevention, Jinan, China; ^3^State Key Laboratory of Pathogen and Biosecurity, Beijing Institute of Microbiology and Epidemiology, Beijing, China; ^4^Global Station for Zoonosis Control, Global Institution for Collaborative Research and Education, Hokkaido University, Sapporo, Japan; ^5^National Virus Reference Laboratory, School of Medicine, University College Dublin, Dublin, Ireland; ^6^Department of Biochemistry and Molecular Biology, Bio21 Molecular Science and Biotechnology Institute, The University of Melbourne, Parkville, VIC, Australia

**Keywords:** Coxsackievirus A4, hand, foot, and mouth disease, genotypes, phylogenetic analysis, *VP1*

## Abstract

Coxsackievirus A4 (CVA4) is one of the most prevalent pathogens associated with hand, foot and mouth disease (HFMD), an acute febrile illness in children, and is also associated with acute localized exanthema, myocarditis, hepatitis and pancreatitis. Despite this, limited CVA4 genome sequences are currently available. Herein, complete genome sequences from CVA4 strains (*n* = 21), isolated from patients with HFMD in Shandong province, China between 2014 and 2016, were determined and phylogenetically characterized. Phylogenetic analysis of the *VP1* gene from a larger CVA4 collection (*n* = 175) showed that CVA4 has evolved into four separable genotypes: A, B, C, and D; and genotype D could be further classified in to two sub-genotypes: D1 and D2. Each of the 21 newly described genomes derived from isolates that segregated with sub-genotype D2. The CVA4 genomes displayed significant intra-genotypic genetic diversity with frequent synonymous substitutions occurring at the third codon positions, particularly within the P2 region. However, *VP1* was relatively stable and therefore represents a potential target for molecular diagnostics assays and also for the rational design of vaccine epitopes. The substitution rate of *VP1* was estimated to be 5.12 × 10^-3^ substitutions/site/year, indicative of ongoing CVA4 evolution. Mutations at amino acid residue 169 in *VP1* gene may be responsible for differing virulence of CVA4 strains. Bayesian skyline plot analysis showed that the population size of CVA4 has experienced several dynamic fluctuations since 1948. In summary, we describe the phylogenetic and molecular characterization of 21 complete genomes from CVA4 isolates which greatly enriches the known genomic diversity of CVA4 and underscores the need for further surveillance of CVA4 in China.

## Introduction

Human enteroviruses (EVs), non-enveloped, single-stranded RNA viruses, are taxonomically classified in the genus *Enterovirus* (Dalldorf, 1953), family *Picornaviridae* and consist of four species: EV-A, EV-B, EV-C, and EV-D (Knowles et al., 2012). Coxsackievirus A4 (CVA4), a member of the EV-A species, is currently composed of 25 types including the common pathogens enterovirus A71 (EVA71) and Coxsackievirus A16 (CVA16) which are the most prevalent agents isolated from pediatric cases of hand-foot-mouth disease (HFMD), a common contagious disease among children which sporadically occur worldwide (Wang et al., 2017).

The CVA4 prototype strain, High Point (GenBank accession number: AY421762), was first isolated from urban sewage during a poliomyelitis outbreak in North Carolina, United States in 1948 (Melnick, 1950; Oberste et al., 2004). In Carey and Myers (1969), another CVA4 strain was isolated from the serum of a child with fatal illness marked by severe central nervous system manifestations. In Estrada Gonzalez and Mas (1977), found that CVA4 was associated with the occurrence of acute polyradiculoneuritis. Notably, CVA4 was also reported as pathogens in HFMD outbreaks (Hu et al., 2011). Associations between CVA4 infection and herpangina (Cai et al., 2015), mucocutaneous lymph node syndrome (Ueda et al., 2015), and bilateral idiopathic retinal vasculitis (Mine et al., 2017) have also been reported. These clinical findings highlight that CVA4 infections exert a disease burden to global public health, especially for neonates and young children which necessitates a greater understanding of the genetic diversity of this pathogen.

Few complete genomes from CVA4 strains were sequenced prior to 2004 and were predominantly from Asia and the United States (Oberste et al., 2004). However, in 2004 and 2006, CVA4 caused two HFMD epidemics in Taiwan (Chu et al., 2011). After 2006, a greater number of CVA4 strains were isolated in Europe and Asia, with the majority from China. For example, seven samples from acute flaccid paralysis (AFP) patients in Shaanxi province collected between 2006 and 2010 were diagnosed with CVA4 infection (Wang et al., 2016). Since 2008, predominantly partial VP1 and a far smaller number of complete genome sequences of multiple CVA4 strains have been reported from clinical specimens from pediatric HFMD cases in mainland China, such as Gansu (Liu et al., 2009), Shenzhen (Hu et al., 2011; Cai et al., 2015), Guangdong (Zhen et al., 2014), and Beijing (Li et al., 2015).

The CVA4 RNA genome (∼7434 nt) can be sub-divided into the 5′-untranslated region (UTR), 3′-UTR, and the open reading frame (ORF) (∼6606 nt) encoding one polyprotein comprising four structural proteins (*VP4, VP2, VP3*, and *VP1* in region P1) and seven non-structural proteins (*2A, 2B*, and *2C* in region P2, and *3A, 3B, 3C*, and *3D* in region P3). The *VP1* gene is typically employed for taxonomic classification of EV types within the genus *Enterovirus* (Palacios et al., 2002). Currently, few full-length genome sequences of CVA4 (*n* = 10) are available in the GenBank database, which limits the understanding of CVA4 genome evolution and necessitates a greater understanding of the genetic diversity in this pathogen to provide an evidence base for the rational development of molecular diagnostics, drug development and vaccine design.

In the present study, we describe the full-length genome sequences (*n* = 21) from CVA4 isolates from pediatric HFMD patients from Shandong province, China identified between 2014 and 2016 and the phylogenetic and molecular characterization of CVA4 *VP1* sequences (*n* = 175). These findings deepen our understanding of CVA4 diversity and evolution and have important implications to mitigate global disease burden attributable to this pathogen.

## Materials and Methods

### Clinical Samples

In this study, stool specimens from children <6 years of age presenting with HFMD were collected between 2014 and 2016, and EV-positive clinical samples which were laboratory-confirmed as non-EVA71 and non-CVA16 infections were kindly provided by the Shandong Center for Disease Control and Prevention.

### CVA4 Isolation and RNA Extraction From Clinical Samples

Human rhabdomyosarcoma (RD) cells were grown in MEM (minimal essential medium, Gibco) supplemented with 10% FBS (fetal bovine serum, Gibco), 50 IU/mL of penicillin and 50 μg/mL of streptomycin. The samples were propagated three times in human RD cells at 37°C in a humid atmosphere under 5% CO_2_.

Total RNA was extracted from the RD supernatant after development of cytopathic effect (CPE) using the RNAiso Plus kit (TaKaRa, 9109). RNA was reverse transcribed into cDNA using random hexamers with the PrimeScript RT Reagent kit (TaKaRa, RR037A). TaqMan-based real-time PCR assays were performed to detect the presence of EV using an ABI 7500 Real-Time PCR System, as previously described (Zhang et al., 2017a,b). The EV positive samples (*n* = 21) were sequenced using the MiSeq high-throughput sequencing platform, and the sequencing data were analyzed using the CLC program to obtain the complete viral genome sequences. Non-EVA71 and non-CVA16 samples from HFMD cases were further tested by a CVA4-specific real-time PCR assay employing oligonucleotide primers and probe designed based on the *VP1* gene of the prototype CVA4 strain (GenBank accession number: AY421762): CVA4-F: TATGGGCTTTGTCCAACTCC, CVA4-R: GTCTAGGGACCCATGCCCTCACT, CVA4-probe: FAM-TGGGGACATTTTCAGCTAGAGTTGTGAGCAAG-BHQ1.

### Phylogenetic Analysis of CVA4

For phylogenetic analysis, two datasets including all available complete genomic sequences (*n* = 10) and the full-length sequences of *VP1* gene (*n* = 154) of CVA4 strains were downloaded from GenBank. Multiple sequence alignments for the two datasets were performed using Muscle (Edgar, 2004), together with the Shandong CVA4 sequenced genomes (*n* = 21). The nucleotide substitution model was chosen using jModeltest (Darriba et al., 2012), and the general time reversible (GTR) model was always the best model for analysis of both datasets. Phylogenetic analysis of the two datasets was conducted using RAxML v8.1.6 (Stamatakis et al., 2005), with the GTRGAMMA nucleotide substitution model with 1000 bootstrap replicates. The nucleotide and amino acid distances were estimated using MEGA 5 (Tamura et al., 2011).

### Genetic Diversity Along the CVA4 Polyprotein Genes

The average pairwise genetic diversity along the CVA4 genomes was calculated using Phylip with a sliding window of 300 nucleotides and a step size of 50 nucleotides (Faria et al., 2017). To identify at which site of the codons that nucleotide variations mostly occur, the *Shannon entropy (Sn)* at positions 1, 2, and 3 of each codon of the aligned polyprotein genes and the *VP4, VP2, VP3, VP1, 2A, 2B, 2C, 3A, 3B, 3C*, and *3D* genes, respectively, were calculated as reported previously (Ramirez et al., 2013). The data processing was performed on the open source R environment (R Development Core Team, 2012), using the ggplot2 package.

### Evolutionary Dynamics of Global CVA4 Strains

To determine the molecular clock-like structure in the CVA4 *VP1* data, the maximum likelihood tree of the *VP1* gene sequences estimated in the previous step was used to study the association between sequence divergence and sampling times using TempEst (Rambaut et al., 2016). To better understand the evolutionary dynamics of CVA4, the *VP1* sequence dataset (*n* = 175) was used to estimate the nucleotide substitution rate using Bayesian Markov chain Monte Carlo (MCMC) sampling implemented in the BEAST v1.8.4 package (Drummond and Rambaut, 2007). The SRD09 model was employed as the nucleotide substitution model. The Bayesian MCMC process was run for 100 million steps, with a sampling frequency of every 5,000 steps and the first 10% of the steps were discarded as burn-in. To select the best combination of the molecular clock and tree prior, both path sampling and stepping-stone sampling (Baele et al., 2012) were employed. The best model combination was a Bayesian skyline tree prior and an uncorrelated relaxed molecular clock model, with log-normally distributed variation in rates among branches (Supplementary Table S1) (Drummond et al., 2005). The posterior distributions were evaluated using Tracer v1.6^1^ where the estimated sample size (ESS) was no less than 200. The Bayesian maximum clade credibility (MCC) tree was generated using TreeAnnotator v1.8.4, and then visualized using FigTree v1.4.3^2^. In addition, we performed a Bayesian skyline plot analysis using Tracer v1.6 program to reconstruct the evolutionary history based on the 175 full-length *VP1* gene sequences of CVA4 strains.

## Results

### Dataset Summary

**FIGURE 1:**
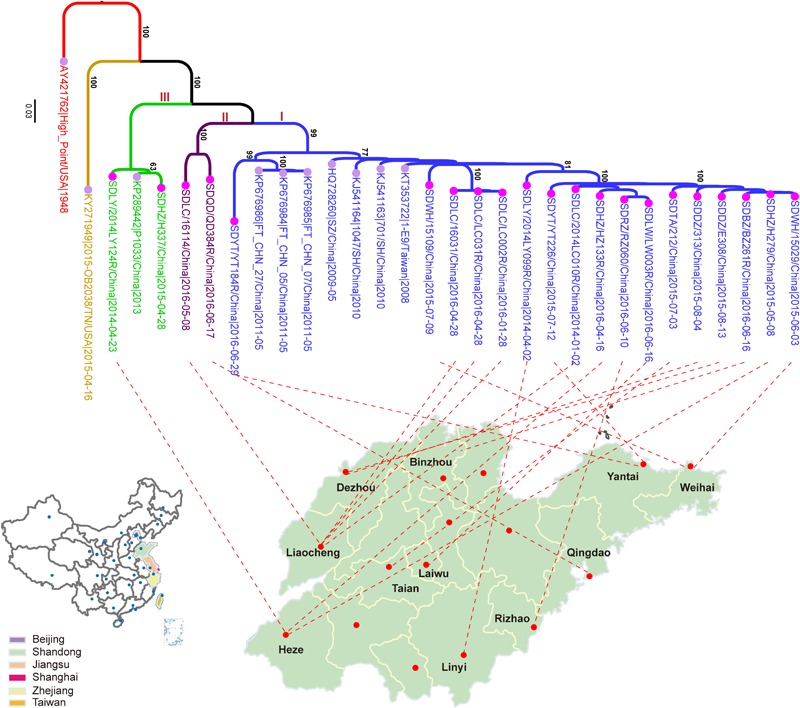
The maximum likelihood phylogenetic tree of the CVA4 whole genome sequences (*n* = 31). The 21 CVA4 isolates described in this study are colored in rose and the nodes of the reference stains downloaded from GenBank are colored in lavender. The three clades (I, II, and III) are colored in blue, purple and light green, and the prototype strain CVA4 High Point (AY421762) is colored in red.

In this study, a total of 21 CVA4 strains were isolated from stool specimens of HFMD patients in Shandong Province, including five from Liaocheng, three from Heze, two each from Dezhou, Linyi, Weihai, Yantai and single samples from Binzhou, Laiwu, Taian, Qingdao and Rizhao (Figure 1 and Supplementary Table S2). The whole genome sequences of the 21 isolates have been deposited into the GenBank database: MH086030-MH086050 (Supplementary Table S2).

Ten complete CVA4 genomes between 1948 and 2015 were publicly available from GenBank, with eight strains from China and two from the United States. Full-length CVA4 *VP1* gene sequences (*n* = 175) strains were also retrieved. They were isolated from the Republic of Azerbaijan (*n* = 1), China (*n* = 145), India (*n* = 8), Kenya (*n* = 1), Russia (*n* = 15), Turkmenistan (*n* = 1), and the United States (*n* = 4) (Supplementary Figure S1), respectively. The collection dates ranged from 1948 to 2016 (Supplementary Figure S1). Notably, 94.9% (*n* = 166) of them were sampled since 2006 and 84.9% (*n* = 141) were collected from 16 provinces and cities in China, including Shandong (*n* = 60), Sichuan (*n* = 18), Beijing (*n* = 14), Shanghai (*n* = 11), Shenzhen (*n* = 9), Hunan (*n* = 7), Yunnan (*n* = 4), Chongqing (*n* = 4), Jilin (*n* = 3), Ningxia (*n* = 2), Jiangsu (*n* = 2), Hainan (*n* = 2), Guangdong (*n* = 2), Anhui (*n* = 1), Henan (*n* = 1), and Zhejiang (*n* = 1).

### Phylogenetic Characterization of CVA4

Phylogenetic analysis of the 31 whole genomes revealed that the 21 isolates from the present study segregated into three strongly supported clades (Figure 1). 17 strains were located in a major clade together with seven Chinese strains collected between 2008 and 2011. Two isolates, SDLC/16114/China/2016-05-08 and SDQD/QD384R/China/2016-06-17 clustered into one independent clade and the isolates SDLY/2014LY124R/China/ 2014-04-23 and SDHZ/H337/China/2015-04-28, as well as the previously described KP289442/P1033/2013/China/2013, belonged to a separable third clade. In addition, the 21 Shandong isolates shared higher nucleotide sequence homologies with eight previously described Chinese strains (82.0–99.8%) than with the prototype strain High Point/United States/1948 (77.8–79.3%) and the American strain, 2015-OB2038/TN/United States/2015-04-16 (75.5–78.3%).

In the maximum likelihood tree of *VP1* genes (Supplementary Figure S2), the CVA4 isolates were divided into four highly supported genotypes: A (*n* = 1), B (*n* = 1), C (*n* = 28), and D (*n* = 145). The prototype High Point strain isolated from the United States in 1948 was classified as genotype A, and one isolate from Kenya in 1999 was designated as genotype B. The CVA4 strains segregating with genotype C were collected from Russia during 2006–2014 (*n* = 14), India in 2010 (*n* = 8), the United States in 1999 (*n* = 1) and 2015 (*n* = 1), Sichuan province, China in 2006 (*n* = 1) and 2007 (*n* = 1), Azerbaijan (*n* = 1) and Turkmenistan (*n* = 1), respectively. Strikingly, genotype D was the predominant genotype in China. Genotype D could be further divided into two highly supported sub-genotypes: D1 (*n* = 5) and D2 (*n* = 140). In detail, five Chinese strains isolated before 2006 belonged to sub-genotype D1, and the remaining Chinese strains clustered within sub-genotype D2, including the 21 isolates described in the present study. However, our sequenced strains did not cluster together, and were interspersed within sub-genotype D2. Notably, there was one CVA4 strain from Russia, KC879539/40238/Russia/2011, falling within sub-genotype D2, and sharing high homology (97.5%) with KY978552/11142/SD/China/2011.

The mean between group nucleotide and amino acid distances were 21.8 and 2.4% (CVA4 genotype A vs. B), 22.5 and 2.8% (A vs. C), 21.7 and 2.3% (A vs. D), 28.9 and 2.6% (B vs. C), 28.0 and 2.9% (B vs. D), and 20.4 and 3.2% (C vs. D), respectively. In addition, the mean nucleotide (and amino acid) distances between groups D1 and D2 was 15.2% (2.2%). The mean nucleotide (and amino acid) distances within groups C and D were 13.2% (2.8%) and 6.0% (1.3%), respectively, i.e., 15%. The mean nucleotide (and amino acid) distances within groups D1 and D2 were 10.4% (2.0%), and 5.3% (1.2%), respectively.

### Pairwise Genetic Diversity Along the CVA4 Genome

**FIGURE 2:**
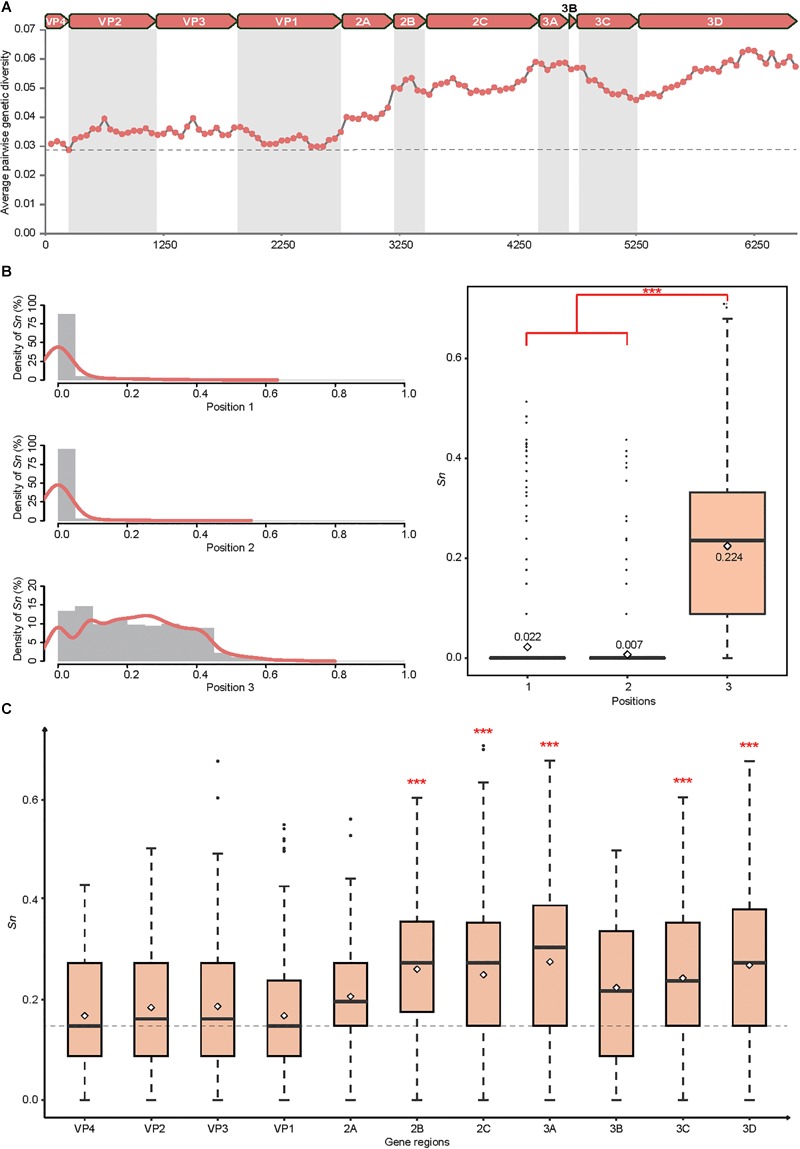
Genetic diversity of the CVA4 polyprotein genes (*n* = 31). **(A)** Average pairwise genetic diversity of all CVA4 polyprotein genes was calculated using a sliding window of 300 nts with a step size of 50 nts. **(B)** Positional entropy values and the *Sn* values at partitions 1, 2, and 3 of each codon were estimated, respectively. ^∗∗∗^*p*-value <0.001 in the Wilcoxon rank-sum test. **(C)** The *Sn* values of nucleotides at positions three of the codons were estimated in *VP4, VP2, VP3, VP1, 2A, 2B, 2C, 3A, 3B, 3C*, and *3D* genes, respectively. ^∗∗∗^*p*-value <0.001 in the Wilcoxon rank-sum test between *2B, 2C, 3A, 3C, 3D* genes and *VP4, VP2, VP3, VP1* genes, respectively.

Employing a sliding windows analysis across CVA4 genomes (*n* = 31), we found that the pairwise genetic diversity at regions P2 (including the *2A, 2B*, and *2C* genes) and P3 (including *3A, 3B, 3C*, and *3D* genes) was higher than that at region P1 (including *VP4, VP2, VP3*, and *VP1* genes) (Figure 2A). In addition, *3A, 3B* and the 3′ terminus of *3D* showed higher pairwise genetic diversity than other gene regions. The polyprotein gene of the 21 CVA4 genomes from the present study had 82.6–99.2% nucleotide sequence homology and a corresponding 97.1–99.9% amino acid sequence homology, suggesting the occurrence of numerous synonymous mutations. Consistent with this observation, the mean *Sn* at the third position of the codons of the polyprotein gene of the CVA4 strains was 0.224, significantly higher than those at positions 1 and 2 of the codons (Figure 2B). Furthermore, the mean *Sn* values at the third position of the codons of *2B, 2C, 3A, 3C*, and *3D* genes were significant higher than those of the *VP1, VP2, VP3*, and *VP4* genes in the region P1 (Figure 2C).

### Evolutionary Dynamics of Worldwide CVA4

**FIGURE 3:**
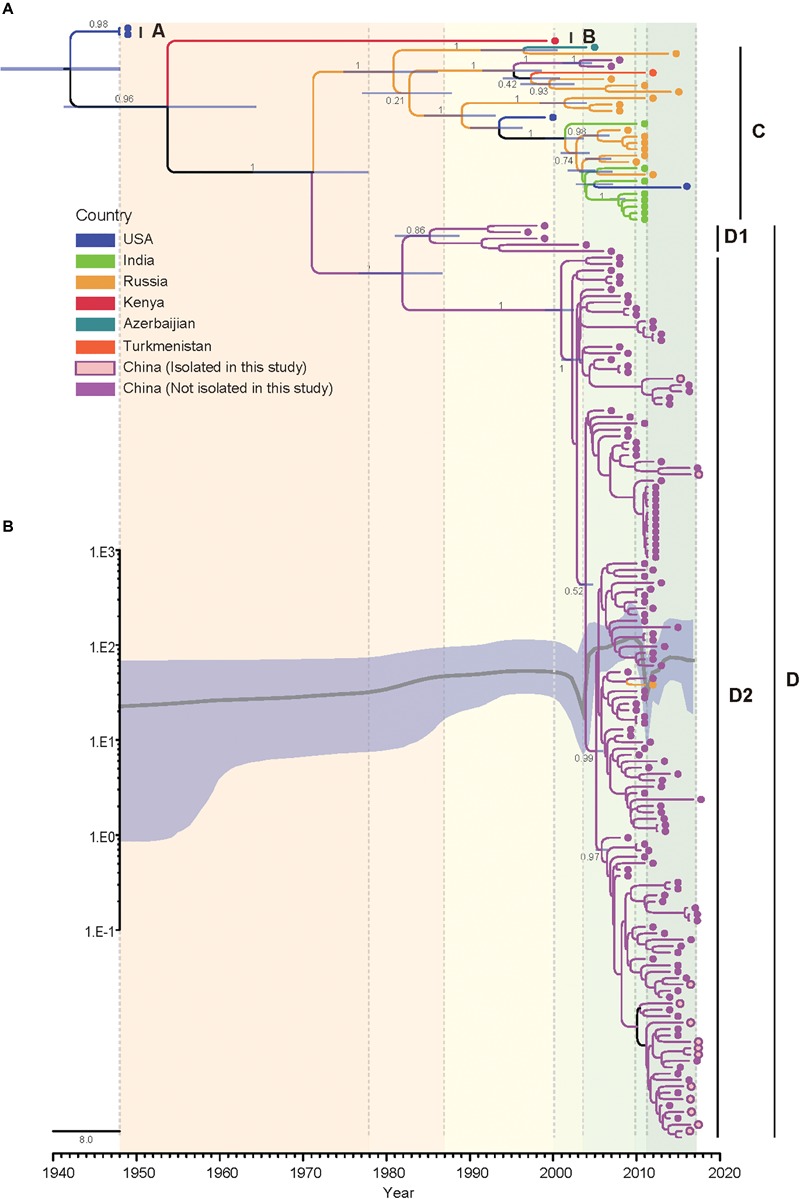
Bayesian phylogenetic analysis and demographic reconstruction of the CVA4 *VP1* gene sequences (915 nts). **(A)** Bayesian phylogeny was conducted by BEAST with a Bayesian skyline tree prior and a relaxed molecular clock model. **(B)** The *x*-axis is the time scale (years) and the *y*-axis is the effective population size in logarithmic Neτ scale. The thick solid line indicates the median estimates and the shaded area indicates the 95% highest posterior density.

The Bayesian phylogenetic tree of all CVA4 *VP1* genes (*n* = 175) reconstructed employing the best-fit parameter model revealed the same topology as the maximum likelihood phylogenetic tree (Figure 3). The Tempest analysis of molecular clock structure revealed a strong temporal correlation in the *VP1* gene (*R*^2^ = 0.86). The mean nucleotide substitution rate for the CVA4 *VP1* genes was estimated to be 5.12 × 10^-3^ substitutions/site/year (95% highest posterior density (HPD): 4.45 × 10^-3^–5.81 × 10^-3^ substitutions/site/year). Around the year 1941 (95% HPD: 1931–1948), the CVA4 viruses were estimated to diverge into genotype A and the common ancestry of genotypes B, C, and D. Genotype B was suggested to have diverged from the common ancestry around 1951 (95% HPD: 1933–1966), and finally genotypes C and the genotype D diverged around 1970 (95% HPD: 1961–1977). Furthermore, genotype D was further divided into sub-genotypes D1 and D2 which occurred approximately in 1979 (95% HPD: 1974–1985), and the time to the most recent common ancestor (tMRCA) of the sub-genotype D2 could be traced back to 2001 (95% HPD: 1999–2003).

Additionally, we performed a Bayesian skyline plot analysis to reconstruct the demographic history of CVA4 based on the *VP1* gene sequences (Figure 3). Our results showed that the effective population size (analog to the number of infected individuals) of CVA4 virus experienced five major stages since the prototype strain High Point was described in 1948 (Figure 3). The first stage covered 1948 to 2000 where the population size was relatively constant, with evidence of a slight increase. During this stage, genotypes B, C, and D were already present before 1975, as well as the emergence of genotype D1 in the early 1980s and the subsequent co-circulation of genotypes B, C, and sub-genotype D1. In the second stage, the population size then experienced a sharp and rapid decrease from 2000 to approximately mid-2003 possibly due to a reduction in the prevalence of genotype B and sub-genotype D1 (or potentially a lack of surveillance data), as genotype C and sub-genotype D2 diversified during this period. Soon after mid-2003, the population size of CVA4 experienced a remarkable and rapid growth until 2010, with the widespread circulation of genotype C in Russia and in India, and sub-genotype D2 in China. After 2010, there was another sharp and abrupt decrease in the effective population size which may have been caused by the decreased prevalence of genotype C. Since 2011, CVA4 appears to have entered a rebound stage with the population size becoming stable again with sub-genotype D2 dominating in China.

### Molecular Characterizations

Compared with the CVA4 prototype strain High Point, 40 amino acid substitutions were identified in the polyprotein in >75% of our isolates, including 1 in *VP4*, 2 in *VP2*, 2 in *VP3*, 6 in *VP1*, 6 in *2A*, 1 in *2B*, 5 in *2C*, 1 in *3A*, 3 in *3C*, and 11 in *3D* (Supplementary Table S3). In EVA71, 14 critical amino acid residues and two gene motifs have been reported previously to be associated with viral infectivity *in vitro* and *in vivo* (Table 1) (30–43). Interestingly, the CVA4 strains pocessed the same amino aicd residues at several sites as those of the EVA71 strain BrCr, such as residues 37, 113, and 192 of the *VP1* protein, 84, 82–86, and 154–156 of the *3C* protein, and 73 and 363 of the *3D* protein (Table 1). However, different from *VP1*_169L_ of the prototype EVA71 strain, *VP1*_169F_ was found in High Point and in each of our 21 CVA4 isolates, indicating that CVA4 may have adapted to bind to the murine scavenger receptor class B member 2 (SCARB2) (Victorio et al., 2016). Notably, there were different amino acid residuals at *VP2*_149_, *VP1*_97_, *VP1*_98_, *VP1*_123_, *VP1*_167_, and *VP1*_244_ between EVA71 BrCr and the CVA4 strains, and *VP1*_145K_ was observed among our CVA4 isolates.

**Table 1:** Comparison of potential critical amino acid residues of the prototype EV71 and CVA4 strains and the 21 isolates described in the present study.

Protein	Amino acid position^a^	EVA71 BrCr (U22521)	CVA4 high point (AY421762)	Shandong isolates (*n* = 21)	Potential biological functions
*VP2*	L149M	K	S	S	Promoting viral binding and RNA accumulation
					Contributes to viral infectivity *in vitro* and mouse lethality *in vivo* (Huang et al., 2012)
	K149I				Alters the tropism in a receptor-dependent or -independent manner (Miyamura et al., 2011)
					Efficient virus replication in Chinese hamster ovary cells (Chua et al., 2008)
*VP1*	H37R	H	H	H	Necessary for K244E rescue in primate cell culture (Caine et al., 2016)
	L97R	L	D	D	Confers ability to use HS as an attachment receptor (Tseligka et al., 2018)
	K98E	K	T	T (*n* = 20), A (*n* = 1)	Confers binding ability to murine SCARB2 (Victorio et al., 2016)
	I113M	I	I	I	Associated with resistance to pocket-binding compounds NLD, GPP3, ALD (Kelly et al., 2015)
	V123I	V	L	L	
	E145A	R	R	L	Confers binding ability to murine SCARB2 (Victorio et al., 2016)
	E145G/Q				Confers binding ability to PSGL-1 (Nishimura et al., 2013) Important residue for mouse adaptation (Zaini and McMinn, 2012)
	E167G	D	E	E	Stabilizing function based on *VP1 3D* structure (Cordey et al., 2012)
	L169F	L	F	F	Confers binding ability to murine SCARB2 (Victorio et al., 2016)
	V192M	V	V	V	Associate with resistance towards BPR0Z-194 (Shih et al., 2004b)
	K244E	E	N	N (*n* = 20), D (*n* = 1)	Important residue for mouse adaptation (Zaini et al., 2012)
					Increased virulence and neuro-tropism in adult interferon-deficient mice (Caine et al., 2016)
*3C*	R84K	R	R	R	Retains good RNA binding and proteolytic activity of the recombinant *3C* (Shih et al., 2004a)
	KFRDI82-86 deletion or QFQ/KNA	KFRDI	KFRDI	KFRDI	Responsible for RNA binding (Shih et al., 2004a)
	VGK154-156T/SAQ	VGK	VGK	VGK	
*3D*	Y73H	Y	Y	Y	Resulting in a strong temperature-sensitive phenotype (Arita et al., 2005)
	C363I	C	C	C (*n* = 20), Y (*n* = 1)	

*^a^Numbering according to EVA71 (U22521).*

## Discussion

Although EVA71 and CVA16 are known as the major causative agents of HFMD in China, a number of other EV-A species including CVA6, CVA10, CVA2, and CVA4 have been reported to be associated with HFMD^3^. In addition, CVA4 was also one of the common pathogens associated with cases of aseptic meningitis, herpetic angina, and viral myocarditis (Carey and Myers, 1969; Estrada Gonzalez and Mas, 1977; Lee et al., 2014; Wang et al., 2016), which has raised serious public health concern and speculation as to whether there is an altered tropism or pathogenicity associated with this CV type. However, detailed genome analysis has been problematic because there were only ten complete CVA4 genomes available in the GenBank database to date (Hu et al., 2011). Therefore, the molecular epidemiological characterization of CVA4 remains far well less understood due to a paucity of available genomic data.

In this study, we have described 21 novel CVA4 genomes isolated from children with HFMD and revealed that our isolates clustered into three highly supported clades in a maximum likelihood tree estimated using the whole genomes. This suggested that there was a certain genetic diversity in the Chinese CVA4 viruses, which would have been underestimated due to the limited surveillance data available. Phylogenetic analysis of the *VP1* gene sequences showed that the CVA4 strains could be classified into four separable genotypes, A-D, with a mean within genotype genetic distance of 0.00–13.2% and the mean between genotype genetic distance of 20.4–28.9%. Therefore, consistent with previous reports (Rico-Hesse et al., 1987; Hu et al., 2011), different CVA4 genotypes could be defined with >15% of between genotype nucleotide distance in the *VP1* gene. However, in contrast with a previous report (Hu et al., 2011), the prototype strain High Point formed an independent branch as genotype A in our classification. The vast majority of the Chinese strains including the 21 isolates in this study belonged to genotype D, with just two strains identified from Sichuan province in 2006 and 2007 belonging to genotype C. In addition, based on the available evidence sub-genotype D2 appears to have supplanted D1 and become predominant in China over the previous decade.

Further analysis showed that mutations were more likely to occur in the P2 and P3 regions encoding non-structural proteins and at the third position of the codons, consistent with previous reports that there was a general synonymous codon usage pattern for many types of enteroviruses (Liu et al., 2011; Ma et al., 2014; Zhang et al., 2014; Su et al., 2017). Moreover, the *VP1* gene was the most conserved region in our analysis, suggesting that it may be a potential target to design a sensitive and CVA4-specific RT-qPCR diagnostic assay and may also be a candidate epitope for development of vaccines.

Since the mid-1950s, the genetic diversity of CVA4 has gradually increased from 1948 to 1999 and experienced a sharp increase between mid-2003 and 2009, paralleling the increased prevalence of CVA4 in China. However, the population size was estimated to have experienced more substantial declines during 1999 and mid-2003 and from 2009 to 2011 than those in a previous report (Chen et al., 2018). Considering that the precision of demographic reconstruction analysis is sensitive to sampling, pinpointing the abrupt decrease of population size of CVA4 between 1999 and mid-2003 would require further sampling efforts. Therefore, national surveillance of various human enteroviruses causing HFMD is urgently needed to elucidate the complex evolutionary dynamics and co-circulation of the enteroviruses to provide an evidence base for rational diagnostic and prophylactic approaches to mitigate disease burden.

The mean evolutionary rate of the CVA4 *VP1* gene was estimated to be 5.12 × 10^-3^ substitutions/site/year, slightly lower but of a similar range to 6.4 × 10^-3^ substitutions/site/year estimated by Chen et al. (2018). We estimated the common ancestor of known CVA4 to have existed approximately 75 years ago, also similar to a previous estimate (71.8 years ago) (Tee et al., 2010). Interestingly, the evolutionary rate of EVA71 *VP1* was estimated to be around 4.6 × 10^-3^ substitutions/site/year for both EVA71 genogroups B and C (Tee et al., 2010; Chen et al., 2018). The evolutionary rate of CVA6 was estimated at 8.1 × 10^-3^ substitutions/site/year (Puenpa et al., 2016), and that of CVA16 was 6.7 × 10^-3^ substitutions/site/year (Zhao et al., 2016). Therefore, CVA4 appears to have been evolving at a similar rate to the other major HFMD pathogens, which deserves further attention.

Coxsackievirus A4 shared phenotype-associated amino acid substitutions when compared to the EVA71 prototype strain, BrCr. Of particular note, the *VP1*_169F_ identified in EVA71 may have conferred viral binding ability to the mammalian SCARB2 receptor, also found in the CVA4 isolates (Yamayoshi et al., 2012). In addition, there were several sites with different amino acid motifs between BrCr and the CVA4 viruses. However, it should be noted that although the biological relevance of these sites with specific amino acid substitutions have been confirmed in EVA71, they were identified through sequence comparison between CVA4 and EVA71, Therefore, the effects of these critical amino acids in CVA4 on receptor binding, viral infectivity, replication mammalian adaptation and pathogenesis warrant further studies.

Additionally, we also detected potential genetic recombination events within the CVA4 genomes (*n* = 31) using the Recombination Detection Program (RDP) v4.16 (Martin et al., 2015) and Simplot v3.5.1 (Hu et al., 2011). However, we failed to detect convincing recombination events in the 21 newly sequenced CVA4 viruses (data not shown). Overall it appears that genetic substitution, rather than recombination, has played a more important role in the evolution and diversification of CVA4, despite recombination events being frequently identified in other human enteroviruses (Hu et al., 2011). However, limited surveillance data may also have led to an underestimation of the number of genetic recombination events on CVA4 evolution.

In summary, we have described 21 novel CVA4 genome sequences, which greatly enriches the available genomic data for CVA4. Phylogenetic analysis revealed that genotypes C, D1 and D2 were co-circulating in the early 2000s and D2 has now supplanted D1 to become the predominant sub-genotype in China. The genetic diversity among Chinese CVA4, occurred at regions encoding the non-structural proteins and at the third wobble position of the codons. In contrast, fewer mutations occurred at the *VP1* gene region, which makes it an optimal candidate region for design of molecular assays and potentially as a conserved epitope for vaccination. Notably, CVA4 may possess higher binding affinity to SCARB2 because of the substitution L169F in the *VP1* protein and how this substitution affects the viral infectivity and clinical outcome should be investigated in the future. In addition to monitoring EVA71 and CVA16, enhanced surveillance of increasingly prevalent and also virulent agents, including CVA4, in China is urgently needed to better understand the dynamics of circulation and evolution of human enteroviruses, which will provide invaluable information for diagnostic and prophylactic approaches to contain disease.

## Author Contributions

MW, JL, and W-FS designed the study, participated in all tests and drafted the manuscript. M-XY, Y-WZ, S-JD, and X-JW participated in collecting and testing samples. TH, MC, SD, X-CZ, Z-JZ, HZ, and Y-GT conceived the study, contributed to the analysis of the results and preparation of revised manuscript versions. All authors read and approved the final manuscript.

## Conflict of Interest Statement

The authors declare that the research was conducted in the absence of any commercial or financial relationships that could be construed as a potential conflict of interest.

## Footnotes

1 http:/beast.bio.ed.ac.uk/Tracer

2 http:/tree.bio.ed.ac.uk/software/figtree/

3 www.picornaviridae.com

## References

[B1] Arita M. Shimizu H. Nagata N. Ami Y. Suzaki Y. Sata T. (2005). Temperature-sensitive mutants of enterovirus 71 show attenuation in cynomolgus monkeys. *J. Gen. Virol.* 86(Pt 5) 1391–1401. 10.1099/vir.0.80784-0 15831951

[B2] Baele G. Lemey P. Bedford T. Rambaut A. Suchard M. A. Alekseyenko A. V. (2012). Improving the accuracy of demographic and molecular clock model comparison while accommodating phylogenetic uncertainty. *Mol. Biol. Evol.* 29 2157–2167. 10.1093/molbev/mss084 22403239 PMC3424409

[B3] Cai C. Yao X. Zhuo F. He Y. Yang G. (2015). [Gene characterization of the VP1 region of Coxsackievirus A4 from herpangina cases in Shenzhen of China]. *Zhonghua Yi Xue Za Zhi* 95 1226–1229. 26081506

[B4] Caine E. A. Moncla L. H. Ronderos M. D. Friedrich T. C. Osorio J. E. (2016). A single mutation in the VP1 of enterovirus 71 Is responsible for increased virulence and neurotropism in adult interferon-deficient mice. *J. Virol.* 90 8592–8604. 10.1128/JVI.01370-16 27440896 PMC5021399

[B5] Carey D. E. Myers R. M. (1969). Isolation of type A4 coxsackie virus from the blood serum of a child with rapidly fatal illness marked by severe central nervous system manifestations. *Indian J. Med. Res.* 57 765–769. 5805379

[B6] Chen P. Wang H. Tao Z. Xu A. Lin X. Zhou N. (2018). Multiple transmission chains of coxsackievirus A4 co-circulating in China and neighboring countries in recent years: phylogenetic and spatiotemporal analyses based on virological surveillance. *Mol. Phylogenet. Evol.* 118 23–31. 10.1016/j.ympev.2017.09.014 28942015

[B7] Chu P. Y. Lu P. L. Tsai Y. L. Hsi E. Yao C. Y. Chen Y. H. (2011). Spatiotemporal phylogenetic analysis and molecular characterization of coxsackievirus A4. *Infect. Genet. Evol.* 11 1426–1435. 10.1016/j.meegid.2011.05.010 21635970

[B8] Chua B. H. Phuektes P. Sanders S. A. Nicholls P. K. McMinn P. C. (2008). The molecular basis of mouse adaptation by human enterovirus 71. *J. Gen. Virol.* 89(Pt 7) 1622–1632. 10.1099/vir.0.83676-0 18559932

[B9] Cordey S. Petty T. J. Schibler M. Martinez Y. Gerlach D. van Belle S. (2012). Identification of site-specific adaptations conferring increased neural cell tropism during human enterovirus 71 infection. *PLoS Pathog.* 8:e1002826. 10.1371/journal.ppat.1002826 22910880 PMC3406088

[B10] Dalldorf G. (1953). The coxsackie virus group. *Ann. N. Y. Acad. Sci.* 56 583–586. 10.1111/j.1749-6632.1953.tb30251.x 13139263

[B11] Darriba D. Taboada G. L. Doallo R. Posada D. (2012). jModelTest 2: more models, new heuristics and parallel computing. *Nat. Methods* 9:772. 10.1038/nmeth.2109 22847109 PMC4594756

[B12] Drummond A. J. Rambaut A. (2007). BEAST: bayesian evolutionary analysis by sampling trees. *BMC Evol. Biol.* 7:214. 10.1186/1471-2148-7-214 17996036 PMC2247476

[B13] Drummond A. J. Rambaut A. Shapiro B. Pybus O. G. (2005). Bayesian coalescent inference of past population dynamics from molecular sequences. *Mol. Biol. Evol.* 22 1185–1192. 10.1093/molbev/msi103 15703244

[B14] Edgar R. C. (2004). MUSCLE: multiple sequence alignment with high accuracy and high throughput. *Nucleic Acids Res.* 32 1792–1797. 10.1093/nar/gkh340 15034147 PMC390337

[B15] Estrada Gonzalez R. Mas P. (1977). Virological studies in acute polyradiculoneuritis, LGBS type. Various findings in relation to Coxsackie A4 virus. *Neurol. Neurocir. Psiquiatr.* 18(2–3 Suppl.) 527–531. 211454

[B16] Faria N. R. Quick J. Claro I. M. Theze J. de Jesus J. G. Giovanetti M. (2017). Establishment and cryptic transmission of Zika virus in Brazil and the Americas. *Nature* 546 406–410. 10.1038/nature22401 28538727 PMC5722632

[B17] Hu Y. F. Yang F. Du J. Dong J. Zhang T. Wu Z. Q. (2011). Complete genome analysis of coxsackievirus A2, A4, A5, and A10 strains isolated from hand, foot, and mouth disease patients in China revealing frequent recombination of human enterovirus A. *J. Clin. Microbiol.* 49 2426–2434. 10.1128/JCM.00007-11 21543560 PMC3147834

[B18] Huang S. W. Wang Y. F. Yu C. K. Su I. J. Wang J. R. (2012). Mutations in VP2 and VP1 capsid proteins increase infectivity and mouse lethality of enterovirus 71 by virus binding and RNA accumulation enhancement. *Virology* 422 132–143. 10.1016/j.virol.2011.10.015 22078110

[B19] Kelly J. T. De Colibus L. Elliott L. Fry E. E. Stuart D. I. Rowlands D. J. (2015). Potent antiviral agents fail to elicit genetically-stable resistance mutations in either enterovirus 71 or Coxsackievirus A16. *Antivir. Res.* 124 77–82. 10.1016/j.antiviral.2015.10.006 26522770 PMC4678291

[B20] Knowles N. J. Hovi T. Hyypiä T. King A. M. Q. Lindberg A. M. Pallansch M. A. (2012). “Picornaviridae,” in *Virus Taxonomy: Classification and Nomenclature of Viruses: Ninth Report of the International Committee on Taxonomy of Viruses* eds King A. M. Q. Adams M. J. Lefkowitz E. Carstens E. B. (San Diego, CA: Elsevier).

[B21] Lee C. J. Huang Y. C. Yang S. Tsao K. C. Chen C. J. Hsieh Y. C. (2014). Clinical features of coxsackievirus A4, B3 and B4 infections in children. *PLoS One* 9:e87391. 10.1371/journal.pone.0087391 24504149 PMC3913601

[B22] Li J. S. Dong X. G. Qin M. Xie Z. P. Gao H. C. Yang J. Y. (2015). Outbreak of febrile illness caused by coxsackievirus A4 in a nursery school in Beijing, China. *Virol. J.* 12:92. 10.1186/s12985-015-0325-1 26084565 PMC4495935

[B23] Liu J. F. Zhang Y. Li H. (2009). [Genetic characterization of VP4-VP2 of two coxsackievirus A4 isolated from patients with hand, foot and mouth disease]. *Zhongguo Yi Miao He Mian Yi* 15 345–349. 20077736

[B24] Liu Y. S. Zhou J. H. Chen H. T. Ma L. N. Pejsak Z. Ding Y. Z. (2011). The characteristics of the synonymous codon usage in enterovirus 71 virus and the effects of host on the virus in codon usage pattern. *Infect. Genet. Evol.* 11 1168–1173. 10.1016/j.meegid.2011.02.018 21382519 PMC7185409

[B25] Ma M. R. Hui L. Wang M. L. Tang Y. Chang Y. W. Jia Q. H. (2014). Overall codon usage pattern of enterovirus 71. *Genet. Mol. Res.* 13 336–343. 10.4238/2014.January.21.1 24535860

[B26] Martin D. P. Murrell B. Golden M. Khoosal A. Muhire B. (2015). RDP4: detection and analysis of recombination patterns in virus genomes. *Virus Evol.* 1:vev003. 10.1093/ve/vev003 27774277 PMC5014473

[B27] Melnick J. L. (1950). Studies on the Coxsackie viruses; properties, immunological aspects and distribution in nature. *Bull. N. Y. Acad. Med.* 26 342–356. 15411584 PMC1929940

[B28] Mine I. Taguchi M. Sakurai Y. Takeuchi M. (2017). Bilateral idiopathic retinal vasculitis following coxsackievirus A4 infection: a case report. *BMC Ophthalmol.* 17:128. 10.1186/s12886-017-0523-2 28724375 PMC5517799

[B29] Miyamura K. Nishimura Y. Abo M. Wakita T. Shimizu H. (2011). Adaptive mutations in the genomes of enterovirus 71 strains following infection of mouse cells expressing human P-selectin glycoprotein ligand-1. *J. Gen. Virol.* 92(Pt 2) 287–291. 10.1099/vir.0.022418-0 20943886 PMC3081077

[B30] Nishimura Y. Lee H. Hafenstein S. Kataoka C. Wakita T. Bergelson J. M. (2013). Enterovirus 71 binding to PSGL-1 on leukocytes: VP1-145 acts as a molecular switch to control receptor interaction. *PLoS Pathog.* 9:e1003511. 10.1371/journal.ppat.1003511 23935488 PMC3723564

[B31] Oberste M. S. Penaranda S. Maher K. Pallansch M. A. (2004). Complete genome sequences of all members of the species Human enterovirus A. *J. Gen. Virol.* 85(Pt 6) 1597–1607. 10.1099/vir.0.79789-0 15166444

[B32] Palacios G. Casas I. Tenorio A. Freire C. (2002). Molecular identification of enterovirus by analyzing a partial VP1 genomic region with different methods. *J. Clin. Microbiol.* 40 182–192. 10.1128/jcm.40.1.182-192.2002 11773114 PMC120085

[B33] Puenpa J. Vongpunsawad S. Osterback R. Waris M. Eriksson E. Albert J. (2016). Molecular epidemiology and the evolution of human coxsackievirus A6. *J. Gen. Virol.* 97 3225–3231. 10.1099/jgv.0.000619 27692044

[B34] R Development Core Team (2012). *R: A Language and Environment for Statistical Computing*. Vienna: R Foundation for Statistical Computing.

[B35] Rambaut A. Lam T. T. Max Carvalho L. Pybus O. G. (2016). Exploring the temporal structure of heterochronous sequences using TempEst (formerly Path-O-Gen). *Virus Evol.* 2:vew007. 10.1093/ve/vew007 27774300 PMC4989882

[B36] Ramirez C. Gregori J. Buti M. Tabernero D. Camos S. Casillas R. (2013). A comparative study of ultra-deep pyrosequencing and cloning to quantitatively analyze the viral quasispecies using hepatitis B virus infection as a model. *Antivir. Res.* 98 273–283. 10.1016/j.antiviral.2013.03.007 23523552

[B37] Rico-Hesse R. Pallansch M. A. Nottay B. K. Kew O. M. (1987). Geographic distribution of wild poliovirus type 1 genotypes. *Virology* 160 311–322. 10.1016/0042-6822(87)90001-8 2821678

[B38] Shih S. R. Chiang C. Chen T. C. Wu C. N. Hsu J. T. Lee J. C. (2004a). Mutations at KFRDI and VGK domains of enterovirus 71 3C protease affect its RNA binding and proteolytic activities. *J. Biomed. Sci.* 11 239–248. 10.1159/000076036 14966374

[B39] Shih S. R. Tsai M. C. Tseng S. N. Won K. F. Shia K. S. Li W. T. (2004b). Mutation in enterovirus 71 capsid protein VP1 confers resistance to the inhibitory effects of pyridyl imidazolidinone. *Antimicrob. Agents Chemother.* 48 3523–3529. 10.1128/AAC.48.9.3523-3529.2004 15328120 PMC514779

[B40] Stamatakis A. Ludwig T. Meier H. (2005). RAxML-III: a fast program for maximum likelihood-based inference of large phylogenetic trees. *Bioinformatics* 21 456–463. 10.1093/bioinformatics/bti191 15608047

[B41] Su W. Li X. Chen M. Dai W. Sun S. Wang S. (2017). Synonymous codon usage analysis of hand, foot and mouth disease viruses: a comparative study on coxsackievirus A6, A10, A16, and enterovirus 71 from 2008 to 2015. *Infect. Genet. Evol.* 53 212–217. 10.1016/j.meegid.2017.06.004 28602802

[B42] Tamura K. Peterson D. Peterson N. Stecher G. Nei M. Kumar S. (2011). MEGA5: molecular evolutionary genetics analysis using maximum likelihood, evolutionary distance, and maximum parsimony methods. *Mol. Biol. Evol.* 28 2731–2739. 10.1093/molbev/msr121 21546353 PMC3203626

[B43] Tee K. K. Lam T. T. Chan Y. F. Bible J. M. Kamarulzaman A. Tong C. Y. (2010). Evolutionary genetics of human enterovirus 71: origin, population dynamics, natural selection, and seasonal periodicity of the VP1 gene. *J. Virol.* 84 3339–3350. 10.1128/JVI.01019-09 20089660 PMC2838098

[B44] Tseligka E. D. Sobo K. Stoppini L. Cagno V. Abdul F. Piuz I. (2018). A VP1 mutation acquired during an enterovirus 71 disseminated infection confers heparan sulfate binding ability and modulates ex vivo tropism. *PLoS Pathog.* 14:e1007190. 10.1371/journal.ppat.1007190 30075025 PMC6093697

[B45] Ueda Y. Kenzaka T. Noda A. Yamamoto Y. Matsumura M. (2015). Adult-onset Kawasaki disease (mucocutaneous lymph node syndrome) and concurrent Coxsackievirus A4 infection: a case report. *Int. Med. Case Rep. J.* 8 225–230. 10.2147/IMCRJ.S90685 26491373 PMC4599061

[B46] Victorio C. B. Xu Y. Ng Q. Meng T. Chow V. T. Chua K. B. (2016). Cooperative effect of the VP1 amino acids 98E, 145A and 169F in the productive infection of mouse cell lines by enterovirus 71 (BS strain). *Emerg. Microbes Infect.* 5:e60. 10.1038/emi.2016.56 27329847 PMC4932649

[B47] Wang D. Xu Y. Zhang Y. Zhu S. Si Y. Yan D. (2016). [Genetic characteristics of coxsackievirus group A type 4 isolated from patients with acute flaccid paralysis in Shaanxi, China]. *Bing Du Xue Bao* 32 145–149. 27396156

[B48] Wang J. Hu T. Sun D. Ding S. Carr M. J. Xing W. (2017). Epidemiological characteristics of hand, foot, and mouth disease in Shandong, China, 2009-2016. *Sci. Rep.* 7:8900. 10.1038/s41598-017-y we performed a Bayesian sk09196-z 28827733 PMC5567189

[B49] Yamayoshi S. Iizuka S. Yamashita T. Minagawa H. Mizuta K. Okamoto M. (2012). Human SCARB2-dependent infection by coxsackievirus A7, A14, and A16 and enterovirus 71. *J. Virol.* 86 5686–5696. 10.1128/JVI.00020-12 22438546 PMC3347270

[B50] Zaini Z. McMinn P. (2012). A single mutation in capsid protein VP1 (Q145E) of a genogroup C4 strain of human enterovirus 71 generates a mouse-virulent phenotype. *J. Gen. Virol.* 93(Pt 9) 1935–1940. 10.1099/vir.0.043893-0 22647370

[B51] Zaini Z. Phuektes P. McMinn P. (2012). Mouse adaptation of a sub-genogroup B5 strain of human enterovirus 71 is associated with a novel lysine to glutamic acid substitution at position 244 in protein VP1. *Virus Res.* 167 86–96. 10.1016/j.virusres.2012.04.009 22575826

[B52] Zhang H. Cao H. W. Li F. Q. Pan Z. Y. Wu Z. J. Wang Y. H. (2014). Analysis of synonymous codon usage in enterovirus 71. *Virusdisease* 25 243–248. 10.1007/s13337-014-0215-y 25674591 PMC4188181

[B53] Zhang Z. Dong Z. Li J. Carr M. J. Zhuang D. Wang J. (2017a). Protective efficacies of formaldehyde-inactivated whole-virus vaccine and antivirals in a murine model of coxsackievirus a10 infection. *J. Virol.* 91:e00333-17. 10.1128/JVI.00333-17 28424287 PMC5469256

[B54] Zhang Z. Dong Z. Wei Q. Carr M. J. Li J. Ding S. (2017b). A neonatal murine model of coxsackievirus A6 infection for evaluation of antiviral and vaccine efficacy. *J. Virol.* 91:e02450-16. 10.1128/JVI.02450-16 28250116 PMC5391469

[B55] Zhao G. Zhang X. Wang C. Wang G. Li F. (2016). Characterization of VP1 sequence of Coxsackievirus A16 isolates by Bayesian evolutionary method. *Virol. J.* 13:130. 10.1186/s12985-016-0578-3 27464503 PMC4963925

[B56] Zhen R. Zhang Y. Xie H. Chen C. Geng J. He P. (2014). [Sequence analysis of VP1 region of coxsackievirus A4 and coxsackievirus A10 in Guangzhou city, 2010-2012]. *Zhonghua Yu Fang Yi Xue Za Zhi* 48 445–450. 25219430

