# Supplementary material for: Genome Analysis of Coxsackievirus A4 Isolates From Hand, Foot, and Mouth Disease Cases in Shandong, China
Source: Front Microbiol. 2019 May 7;10:1001. doi: 10.3389/fmicb.2019.01001 (PMC6513881; doi:10.3389/fmicb.2019.01001)
Supplement: Supplementary file 1 [file Table_1.DOC]

**Supplementary Table S1 |** Logarithm of marginal likelihood estimation (logMLE) calculated using stepping-stone sampling and path sampling methods for different demographic models.

| Clock Model | Tree Prior | Using stepping-stone sampling | Using path sampling |
| --- | --- | --- | --- |
| **Relaxed** | **Skyline** | **-15639.58** | **-15635.41** |
|  | Constant | -15655.13 | -15653.84 |
|  | SkyGrid | -15651.81 | -15650.69 |
|  | Skyride | -15698.91 | -15704.91 |
|  | Exponential | -15654.58 | -15652.68 |
| **Stirct** | Skyline | -15663.40 | -15661.56 |
|  | Constant | -15679.32 | -15677.57 |
|  | SkyGrid | -15682.43 | -15679.37 |
|  | Skyride | -15750.23 | -15744.54 |
|  | Exponential | -15663.32 | -15661.50 |
