# Supplementary material for: Genome Analysis of Coxsackievirus A4 Isolates From Hand, Foot, and Mouth Disease Cases in Shandong, China
Source: Front Microbiol. 2019 May 7;10:1001. doi: 10.3389/fmicb.2019.01001 (PMC6513881; doi:10.3389/fmicb.2019.01001)
Supplement: Supplementary file 2 [file Table_2.doc]

**Supplementary Table S2 |** Background information of the 21 clinical CVA4 isolates in this study and the 10 reference strains with complete genome sequences available in Genbank

|  | Accession no. | Strain | Collection location | Collection date | Classification based on the whole genome | Genotype classification based on the *VP1* gene |
| --- | --- | --- | --- | --- | --- | --- |
| Our isolates | MH086030 | SDHZ/H337/China/2015 | Heze | 2015/4/28 | I | D2 |
|  | MH086031 | SDLY/2014LY124R/China/2014 | Linyi | 2014/4/23 | I | D2 |
|  | MH086032 | SDYT/YT184R/China/2016 | Yantai | 2016/6/29 | I | D2 |
|  | MH086033 | SDLC/LC002R/China/2016 | Liaocheng | 2016/1/28 | I | D2 |
|  | MH086034 | SDLC/16031/China/2016 | Liaocheng | 2016/4/28 | I | D2 |
|  | MH086035 | SDLC/LC031R/China/2016 | Liaocheng | 2016/4/28 | I | D2 |
|  | MH086036 | SDWH/15109/China/2015 | Weihai | 2015/7/9 | I | D2 |
|  | MH086037 | SDLY/2014LY099R/China/2014 | Linyi | 2014/4/2 | I | D2 |
|  | MH086038 | SDLW/LW003R/China/2016 | Laiwu | 2016/6/16 | I | D2 |
|  | MH086039 | SDDZ/313/China/2015 | Dezhou | 2015/8/4 | I | D2 |
|  | MH086040 | SDYT/YT226/China/2015 | Yantai | 2015/07/12 | I | D2 |
|  | MH086041 | SDTA/212/China/2015 | Taian | 2015/7/3 | I | D2 |
|  | MH086042 | SDDZ/E308/China/2015 | Dezhou | 2015/8/13 | I | D2 |
|  | MH086043 | SDBZ/BZ261R/China/2016 | Binzhou | 2016/6/16 | I | D2 |
|  | MH086044 | SDWH/15029/China/2015 | Weihai | 2015/6/3 | I | D2 |
|  | MH086045 | SDHZ/H279/China/2015 | Heze | 2015/5/8 | I | D2 |
|  | MH086046 | SDLC/2014LC010R/China/2014 | Liaocheng | 2014/1/2 | I | D2 |
|  | MH086047 | SDHZ/HZ133R/China/2016 | Heze | 2016/4/16 | I | D2 |
|  | MH086048 | SDQD/QD384R/China/2016 | Qingdao | 2016/6/17 | II | D2 |
|  | MH086049 | SDLC/16114/China/2016 | Liaocheng | 2016/5/8 | II | D2 |
|  | MH086050 | SDRZ/RZ060R/China/2016 | Rizhao | 2016/6/10 | III | D2 |
| Reference strains with whole genomes | KP289442 | P1033/China/2013 | China | 2013 | III | D2 |
|  | KP676986 | FT_CHN_27/China/2011 | China | 2011-05 | I | D2 |
|  | KP676984 | FT_CHN_05/China/2011 | China | 2011-05 | I | D2 |
|  | KP676985 | FT_CHN_07/China/2011 | China | 2011-05 | I | D2 |
|  | HQ728260 | SZ/China/2009-05 | China | 2009-05 | I | D2 |
|  | KJ541164 | 1047/SH/China/2010 | China | 2010 | I | D2 |
|  | KJ541163 | 701/SH/China/2010 | China | 2010 | I | D2 |
|  | KT353722 | 1-E9/Taiwan/2008 | Taiwan | 2008 | I | D2 |
|  | KY271949 | 2015-OB2038/TN/USA/2015 | USA | 2015-04-16 |  | C |
|  | AY421762 | High_Point/USA/1948 | USA | 1948 |  | A |
