# Supplementary material for: Genome Analysis of Coxsackievirus A4 Isolates From Hand, Foot, and Mouth Disease Cases in Shandong, China
Source: Front Microbiol. 2019 May 7;10:1001. doi: 10.3389/fmicb.2019.01001 (PMC6513881; doi:10.3389/fmicb.2019.01001)
Supplement: Supplementary file 3 [file Table_3.DOC]

**Supplementary Table S3 |** Comparison of amino acid substitutions between the prototype strain CVA4 and the 21 isolates described in this study.

| Protein | Amino Acid Position ^a^ | CVA4 High Point (AY421762) | Shandong isolates |
| --- | --- | --- | --- |
| VP4 | 47 | T | 95.45%S, 4.55%T |
| VP2 | 143 | N | 95.45%D, 4.55%N |
|  | 163 | V | 81.82%T, 9.09%A, 4.55%I, 4.55%V |
| VP3 | 33 | T | 86.36%V, 9.09%A, 4.55%T |
|  | 65 | N | 86.36%S, 13.64%N |
| VP1 | 23 | T | 95.45%V, 4.55%T |
|  | 34 | A | 77.27%T, 22.73%A |
|  | 102 | S | 95.45%A, 4.55%S |
|  | 200 | A | 72.73%T, 22.73%A |
|  | 262 | I | 95.45%V, 4.55%I |
|  | 285 | Y | 95.45%H, 4.55%Y |
| 2A | 10 | I | 86.36%V, 13.64%I |
|  | 37 | A | 90.91%S, 4.55%T, 4.55%A |
|  | 68 | R | 86.36%K, 13.64%R |
|  | 82 | V | 95.45%I, 4.55%V |
|  | 83 | F | 95.45%Y, 4.55%F |
|  | 102 | E | 59.09%Q, 27.27%V, 13.64%E |
| 2B | 33 | M | 86.36%I, 9.09%M, 4.55%L |
| 2C | 37 | V | 86.36%I, 13.64%V |
|  | 75 | A | 72.73%V, 27.27%A |
|  | 103 | T | 90.91%A, 9.09%T |
|  | 140 | S | 95.45%G, 4.55%S |
|  | 142 | V | 95.45%I, 4.55%V |
| 3A | 8 | I | 77.27%V, 22.73%I |
| 3C | 56 | I | 86.36%V, 13.64%I |
|  | 93 | N | 86.36%S, 13.64%N |
|  | 98 | N | 95.45%S, 4.55%N |
| 3D | 22 | H | 77.27%R, 22.73%H |
|  | 37 | D | 77.27%N, 22.73%D |
|  | 91 | N | 90.91%D, 9.09%N |
|  | 93 | S | 86.36%T, 13.64%S |
|  | 105 | E | 90.91%D, 9.09%E |
|  | 140 | K | 72.73%R, 27.27%K |
|  | 165 | V | 72.73%I, 13.64%V, 9.09%M, 4.55%L |
|  | 190 | V | 86.365A, 9.09%V, 4.55%T |
|  | 216 | T | 86.36%V, 13.64%T |
|  | 315 | R | 81.82%K, 13.64%R |
|  | 346 | R | 72.73%K, 27.27%R |

^a^ All CVA4 genes employ CVA4 numbering.
