# Supplementary material for: Genome Analysis of Coxsackievirus A4 Isolates From Hand, Foot, and Mouth Disease Cases in Shandong, China
Source: Front Microbiol. 2019 May 7;10:1001. doi: 10.3389/fmicb.2019.01001 (PMC6513881; doi:10.3389/fmicb.2019.01001)

Supplemental Figure S1 | Spatio-temporal distribution of the *VP1* gene sequences from CVA4 strains available in GenBank. (A) Collection date; (B) Collection location.

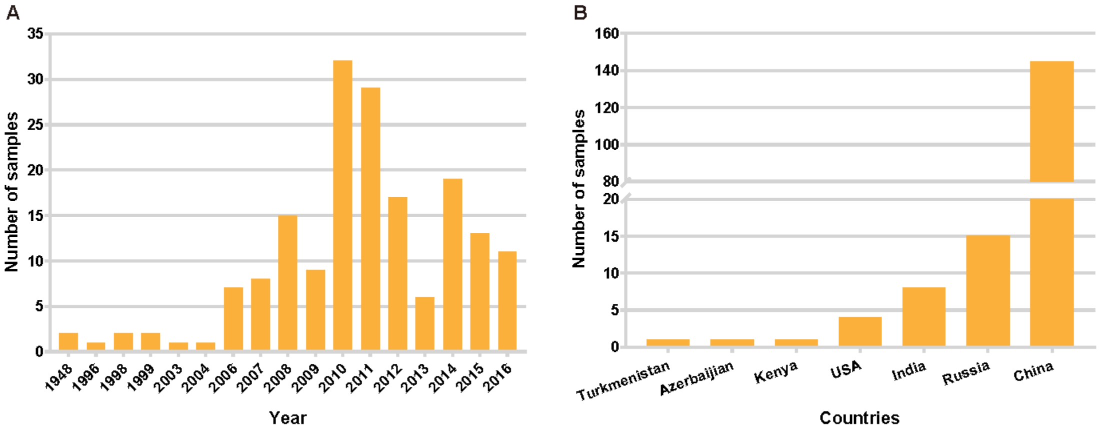

Supplement: Supplementary file 4 [file Data_Sheet_1.pdf]
