# Supplementary material for: Genome Analysis of Coxsackievirus A4 Isolates From Hand, Foot, and Mouth Disease Cases in Shandong, China
Source: Front Microbiol. 2019 May 7;10:1001. doi: 10.3389/fmicb.2019.01001 (PMC6513881; doi:10.3389/fmicb.2019.01001)

Supplemental Figure S2 | The maximum likelihood phylogenetic tree of the complete CVA4 *VP1* gene (915 nts) sequences constructed using RAxML v8.1.6.

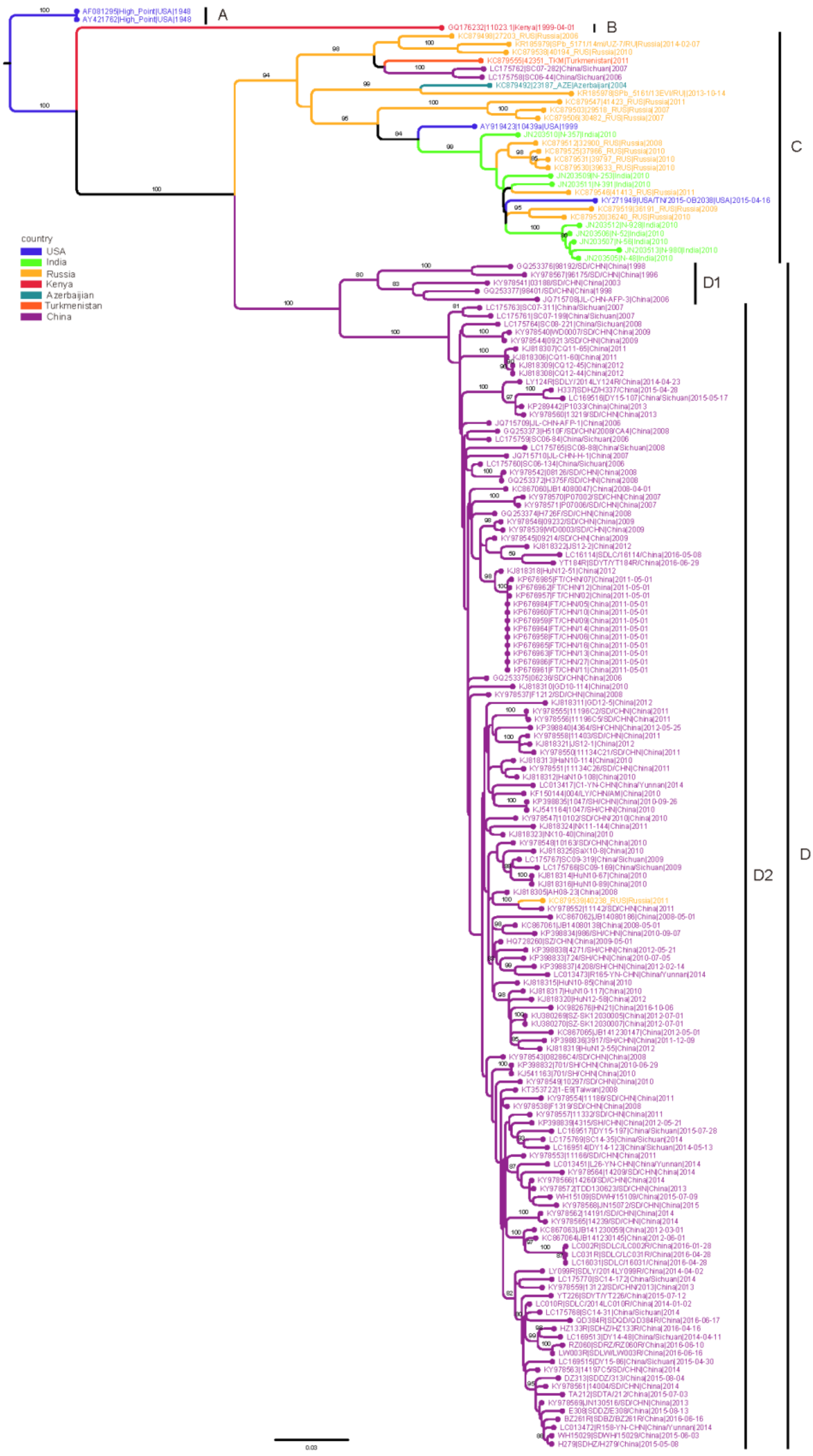

Supplement: Supplementary file 5 [file Data_Sheet_2.pdf]
